# The British Medical Benevolent Fund and Guild

**Published:** 1910-01-01

**Authors:** Adeline M. Bedford, Katherine Westminster, E. Broadbent, May Thorne, R. Douglas Powell, T. Henry Butlin, William S. Church, Thomas Barlow, John Tweedy, Samuel West


*To the Editor of* The Hospital.

### THE BRITISH MEDICAL BENEVOLENT FUND AND GUILD.

*To the Editor of* The Hospital.

Sir,—The tragic fate of Dr. Wells, of St. Mary's Hospital, whose death last October, at the age of thirty years, was caused by contracting glanders in the exercise of his duties, aroused the sympathy of the public and has drawn attention to the dangers to which all medical men are exposed in the pursuit of their profession. Accident or disease may cut short the career of a medical man, or other adverse circumstances may prevent him from making any provision for old age, so that he himself and those dependent upon him may be left in actual want and even encumbered with debt. Speaking at a meeting in support of the Fund held at the Royal College of Physicians in February last, the Bishop of Oxford said: "I do not think there is any profession in which a man is compelled, without any choice of his own, without any chance of escape, to run so great a risk of inevitable impoverishment for himself and for his family. He really risks his all upon his health.

To meet such contingencies the British Medical Benevolent Fund was instituted in the year 1836 (1) to make grants of money to distressed members of the medical profession, their widows or orphans, and (2) to provide annuities for such after they have reached the age of sixty years.

During these 73 years the Fund has expended £70,000 in grants, and rather more than this sum in annuities. At the present time the Fund is supporting 126 annuitants, who receive from £20 to £26 a year each, and in addition makes grants to others not yet eligible for annuities, spending in this way an aggregate of nearly £5,000 a year, the working expenses being under 6 per cent. Yet the demands upon the Fund far exceed its resources, and much-needed help has frequently to be denied to applicants approved by the committee.

A Woman's Guild has recently been established in connection with the Fund to supplement the money grants of the Fund by gifts of clothing and coals and other additional comforts, and by personal service to add a warmer touch of human sympathy.

At the meeting above referred to, the Lord Mayor of London, after drawing attention to the economic value of the services of the profession to the State and to the community, whether in general practice or in hospitals, or in the departments of public health and preventive medicine, asked: "What has the State and what has the public done in return?" "I think, very little," he added, "and it appears to me that the public requires calling to its duties in this respect."

The Fund gives money which has hitherto been mainly supplied by the profession itself; the Guild will give money's worth. For Fund and Guild alike increased support is needed, and in behalf of both we plead and fain would trust that the public will not fail to recognise its obligation to the medical profession by a liberal response to the appeal which we now make.

Subscriptions and donations for the Fund may be sent to the Treasurer of the Fund, 15 Wimpole Street, and for the Guild to the Secretary of the Guild, 100 Harley Street, by both of whom further information will gladly be supplied.

| For the Guild: | For the Fund: |
|---|---|
| Adeline M. Bedford, | R. Douglas Powell, |
| Katherine Westminster, | T. Henry Butlin, |
| E. Broadbent, | William S. Church, |
| May Thorne. | Thomas Barlow, |
| | John Tweedy, |
| | Samuel West. |

